# Supplementary material for: A Probabilistic Functional Atlas of Human Occipito-Temporal Visual Cortex
Source: Cereb Cortex. 2020 Sep 24;31(1):603–19. doi: 10.1093/cercor/bhaa246 (PMC7727347; doi:10.1093/cercor/bhaa246)
Supplement: Supplemental_Materials_bhaa246 [file supplemental_materials_bhaa246.docx]

**Supplemental Materials**

Mona Rosenke^1*^, Rick van Hoof^2*^, Job van den Hurk^2,3^, Kalanit Grill-Spector^1,4^, Rainer Goebel^2^

1 Department of Psychology. Stanford University, Stanford. CA. USA

2 Department of Cognitive Neuroscience, Faculty of Psychology and Neuroscience, Maastricht University, Maastricht, The Netherlands

3 Scannexus MRI Center, Maastricht, The Netherlands

^4^ Wu Tsai Neurosciences Institute, Stanford University

*authors contributed equally

**Corresponding Author:**

Rainer Goebel, Department of Cognitive Neuroscience, Faculty of Psychology and Neuroscience, Maastricht, The Netherlands, r.goebel@maastrichtuniversity.nl


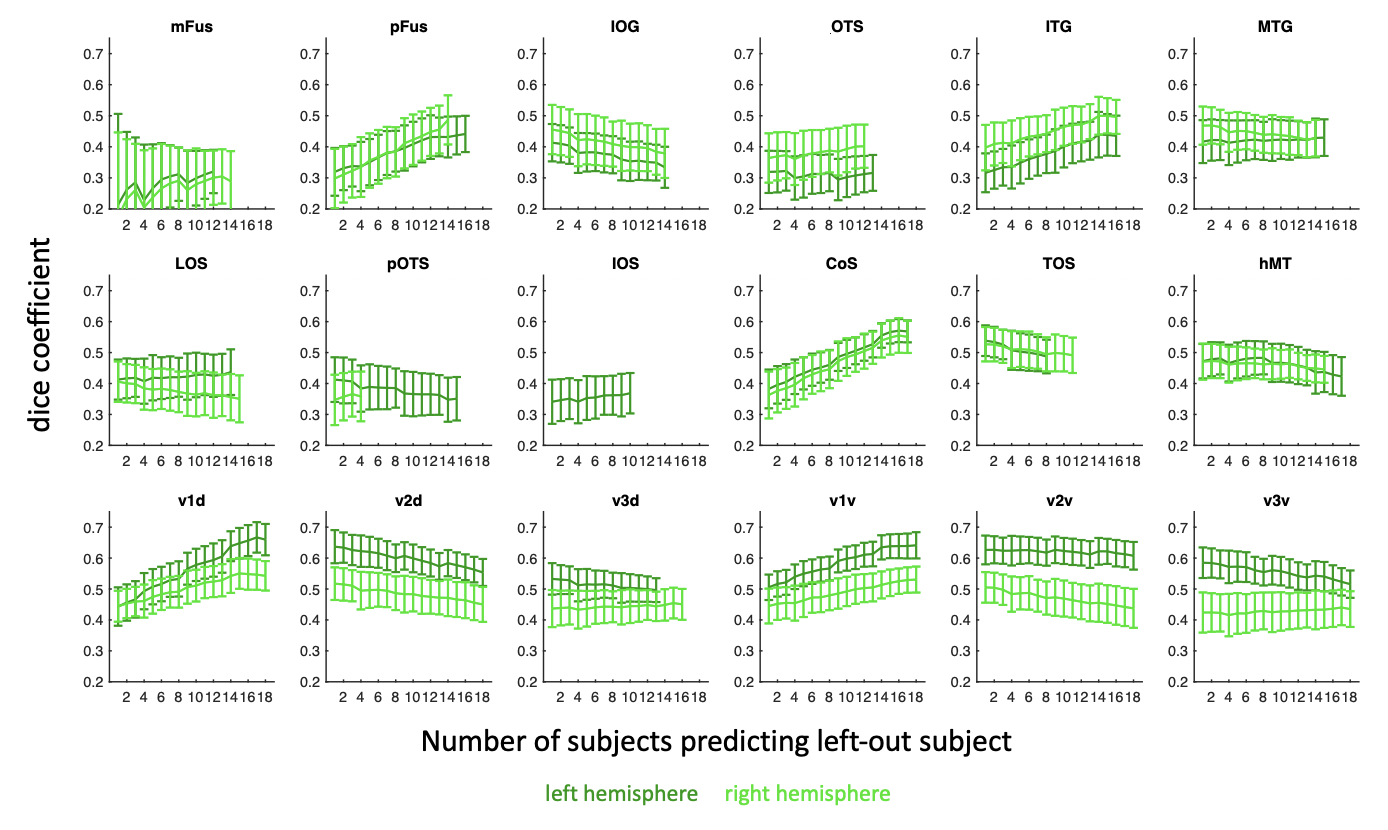


Supplemental Figure 1***.*** **Effect of number of subjects in a group map on the Dice coefficient for predicting left-out subjects’ fROIs.** For each iterative number of subjects comprising an atlas we tested how well it predicts a left-out data using the Dice coefficient metric. x-axis: number of subjects predicting a left-out subject; y-axis: resulting Dice coefficients. *Errorbars:* standard deviation across 1000 sample computations.

**Methodological approach**

To evaluate the effect of number of subjects on the Dice coefficient for a given fROI, we calculated the Dice coefficient with an iterative number of subjects comprising the predicting group maps for each fROI in the visfAtlas. Details for the number of subjects that each fROI was defined in can be found in **Table 1** of the main article. For each fROI and hemisphere, respectively, we started with N = 2 subjects where 1 subject was used to predict the other subject. Then we randomly, without replacement, drew N = 3 subjects and used two to predict the third subject. The prediction was quantitively evaluated with the Dice coefficient. For any predicting number of subjects and each fROI, we used the same threshold that was best across all alignment methods (see main article, Methods and Materials), which was 0.2. Within each iteration of a given number of subjects, we cross-validated the dice coefficient for each left-out subject so that there were three different cross-validation iterations for N = 3, since each subject was left out once. The number of subjects was increased until the total N for the respective hemisphere ROI was reached (see x-axis of Suppl. Fig. 1). Next, we repeated this procedure for each N 1000 times (where the same subjects could not be drawn within the same sample, but could for any of the 1000 times) and computed the standard deviation across those iterations. We chose to draw samples 1000 times to control for the fact that there are more possible combinations of lower N than higher N.
